# Managing laboratory automation in a changing pharmaceutical industry

**DOI:** 10.1155/S1463924695000101

**Published:** 1995

**Authors:** Michael L. Rutherford

**Affiliations:** Quality Control Laboratories Eli Lilly and Company Lilly Corporate Center Indianapolis IN 46285 USA

## Abstract

The health care reform movement in the USA and increased requirements by regulatory agencies continue to have a major impact on the pharmaceutical industry and the laboratory. Laboratory management is expected to improve effciency by providing more analytical results at a lower cost, increasing customer service, reducing cycle time, while ensuring accurate results and more effective use of their staff. To achieve these expectations, many laboratories are using robotics and automated work stations. Establishing automated systems presents many challenges for laboratory management, including project and hardware selection, budget justification, implementation, validation, training, and support. To address these management challenges, the rationale for project selection and implementation, the obstacles encountered, project outcome, and learning points for several automated systems recently implemented in the Quality Control Laboratories at Eli Lilly are presented.


Journal of Automatic Chemistry, Vol. 17, No. 2 (March-April 1995), pp. 59-63

### Managing laboratory automation in a changing pharmaceutical industry

**Michael L. Rutherford**
*Quality Control Laboratories, Eli Lilly and Company, Lilly Corporate Center, Indianapolis, IN 46285, USA*

*The health care reform movement in the USA and increased requirements by regulatory agencies continue to have a major impact on the pharmaceutical industry and the laboratory. Laboratory management is expected to improve efficiency by providing more analytical results at a lower cost, increasing customer service, reducing cycle time, while ensuring accurate results and more effective use of their staff. To achieve these expectations, many laboratories are using robotics and automated work stations. Establishing automated systems presents many challenges for laboratory management, including project and hardware selection, budget justification, implementation, validation, training, and support. To address these management challenges, the rationale for project selection and implementation, the obstacles encountered, project outcome, and learning points for several automated systems recently implemented in the Quality Control Laboratories at Eli Lilly are presented.*

### Introduction

The health care reform movement and increased regulatory requirements continue to have a major impact on the pharmaceutical industry. Changing customer bases, combined with more emphasis on lowering costs and a demand for more accurate data are greatly affecting the way the industry does business, both internally and externally. The pharmaceutical industry is being forced to become more efficient and cost effective to remain competitive in this changing market. As a result, laboratory management is expected to improve efficiency by providing more analytical results at a lower cost, increasing customer service, reducing cycle time, while ensuring accurate results and more effective use of their staff. To achieve these expectations, many laboratories are using robotics and automated work stations. Automation has been utilized to improve efficiency and productivity, reduce cycle times, improve technical staff utilization, provide a safer work environment, and improve the quality of analytical results. However, establishing automated systems in the laboratory present many challenges for laboratory management, including project and hardware selection, budget justification, implementation, validation, training, and support.

### Project and hardware selection

Project selection is the first major challenge for laboratory management, and, in many cases, can be a key factor in whether a project fails or succeeds. It can also have an impact on the establishment of an automation program and future of automation projects in an area. Automation has typically been utilized to improve efficiency and productivity, better utilize technical staff, provide a safer work environment, and improve the quality of analytical results. The first task is to identify potential opportunities for automation. This can be accomplished in several ways. One approach is to identify available technology and identify applications that may be able to utilize this technology. This can be quite successful for work stations when just starting to establish automation in a laboratory. However, this may not address specialized needs or applications where the benefits of automation may be greater. The opposite approach is to identify tasks or assays that can benefit from automation and then identify technology that can address these needs. This approach usually is more amenable to custom automation projects and results in better project selection and utilization of technology.

One of the best ways we have found to identify opportunities is to combine these two approaches using a cross-functional team of technical and automation experts from such areas as the laboratories, systems, engineering, development, and management. Discussions are held with laboratory analysts to identify those assays and/or tasks that are labour intensive, time consuming, high volume, repetitive, highly variable, result in unstable intermediates, or utilize compounds, solvents, reagents or techniques that present a health or safety risk. Other factors that should also be discussed are work flow through the laboratory, assay turn-around needs, laboratory space constraints, technical level of the staff and support groups, and openness to new technology. This approach has been used several times to date, both in Indianapolis and in at least one affiliate, and has helped identify numerous automation and/or productivity improvement opportunities.

Once potential opportunities are identified, final selection of a project or projects is the next critical step. Business needs and drivers most often will impact the final decision. In the pharmaceutical industry, improving efficiency, lowering costs, increasing customer service, reducing cycle time, and effective use of staffs are key drivers when assessing benefits of undertaking a project. Other business drivers to consider are the availability of the necessary resources to implement projects, availability of project capital, and time constraints, which impact the ability to implement a project. Almost always, the decision will hinge on the benefits observed versus the cost or ease of implementing the project. Evaluating the benefits versus the ease of implementation can be accomplished in several ways, and each company has its process for making this decision. One tool that is beneficial is an opportunity map, shown in figure 1.

This paper was presented at the 1994 ISLAR meeting.

© Zymark Corporation 1995

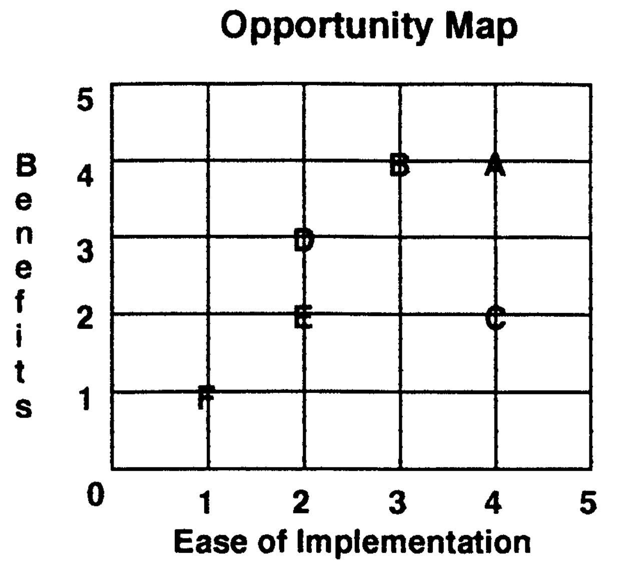

*Figure 1. Opportunity map for project selection.*

This tool was first used as part of a quality/performance improvement program implemented at Lilly in the late 1980s known as 'Performance Excellence. To develop the opportunity map, each project was evaluated solely on its benefits, assigning a value zero to five, with five being high benefit and zero being low benefit. Similarly, each project was evaluated solely on its ease of implementation, again assigning a value of zero to five, with five being easily implemented and zero being difficult to implement. For example, in figure 1, project A, would have a benefit value of four (high benefit) and an ease of implementation value of four (easily implemented). This project might correlate to an application using readily available technology, requiring minimal training of analysts, can be started up with minimal effort, and will reduce cycle time, headcount, etc. in the laboratory. Those projects in the upper right quadrant would be projects easily implemented that would provide the most benefit. The projects would be rank ordered, based on the diagonal as you move from upper right to lower left. In the example, this would be Projects A, B, C, D, E, F. This evaluation of benefits and ease of implementation can be very data driven based on a thorough evaluation of technology, staffing etc., or based more on available data and knowledge of the project scope. The latter approach typically is used when a large number of opportunities have been identified and the objective is to effectively reduce the list to a manageable number of projects prior to doing an in depth evaluation of each project.

Hardware evaluation and/or selection can be incorporated into the project selection process at various places. Some knowledge of available hardware, hardware vendors, and the services they can provide is important throughout the selection process, especially when assessing the ease of implementation. Hardware selection can be affected by several factors. The first to consider is available space for automated equipment—limited space for laboratories is becoming more and more of an issue for laboratory management. Bench top workstations, such as the Zymark Benchmate or Benchmate Tablet Processing Workstation (TPW) (Zymark Corporation, Hopkinton, MA), require minimal space and/or laboratory modifications. However, the trade off is usually limited programming flexibility and customization capabilities. Full size robotics options, such as the Zymate (Zymark Corporation, Hopkinton, MA) or Mitsubishi Micro Robot (Mitsubishi Electronics Corp., Japan) can require significantly more space and typically require laboratory modifications, but can be fully customized and provide greater flexibility.

A second factor is the availability and capability of the hardware itself. Usually several vendors can provide hardware to meet customers' needs, some better than others. For example, some vendors can supply not only the robotics arm, but also complete systems and/or many of the integrated components, such as power and event controllers, centrifuges, liquid handling, racks etc. One very important aspect of hardware selection is to choose the hardware that is best suited for the task. Physical specifications can influence this decision. For example, the load capacity of the robotics arm was a factor in hardware selection for a feed assay application (Hinshaw *et al.* [1]) in our laboratory due to large sample volume and container requirements. Programming and system flexibility can also impact the selection of hardware, depending on the application. Many times this is true for workstations, which tend to be less flexible, limiting their suitability for more specialized applications. Workstations typically do, however, offer the flexibility of being used for multiple assays or tasks very easily—this is not always possible for larger robotics systems which tend to be more dedicated to a specific task or methodology. Flexibility can be built into these systems, but it usually increases the complexity of the programming and the system.

The complexity of the project can be also affected by the selection of hardware. This is another factor that needs to be considered. Some vendors' hardware is better suited for certain applications than others due to ease of programming, availability of integrated components, design, etc. For example, using the Mitsubishi Micro Robot for automated dissolution testing is feasible, but the complexity of this system would be greatly increased. A Zymate-based automated dissolution system would result in a less complex system due to the availability of the components from Zymark and their previous experience with this application.

The vendor is also a factor when selecting hardware. More and more vendors are getting involved in laboratory automation, workstations, and systems integration, making hardware selection even more difficult. Each vendor offers a wide variety of hardware, services, and support which can differentiate them from one another, as well as be very beneficial to the laboratory manager. Systems integration, custom systems and components, and validation services are a few examples of services that can make a difference in hardware selection when multiple options exist. The vendor also has a big influence on another hardware selection factor—cost. In the pharamaceutical industry, more and more emphasis is being placed on reducing manufacturing costs. As a result, laboratory management is expected to improve laboratory productivity and efficiency by providing more analytical results at a lower cost with less staff. The cost versus benefits gained of automated systems and hardware can most often be the biggest factor for resourcing and funding a project.

The last factors in hardware selection are expertise and support. External expertise and support from the vendor is important, but to be truly efficient and effective, it is even more important to have these skills internally. Standardization on a few types of hardware is beneficial since it allows for the development of internal expertise and the necessary support functions. However, it is important not to let this be the primary factor in selecting hardware, especially when better options exist.

### Budget justification

Once a project has been selected and the hardware options evaluated, the next major challenge is the budget justication. Automated systems can be justified in many ways. One of the more common ways is through efficiency and productivity gains. These gains can be based on increased sample capacity, absorption of additional workload, more efficient processing of samples, increased utilization of equipment etc. Based on the cost savings for each of these gains, one should be able to estimate the Return on Investment, or ROI, for the proposed system. This is another way of justifying the purchase on an automated system. Typically, an ROI of two years or less is considered good, but varies from company to company. For example, purchase of one of our Benchmate TPW's was based on the ability to increase our sample capacity for several assays. When the ROI was estimated, it showed that the system would pay for itself in six to nine months based purely on the analysts hours saved. It also allowed us to absorb some additional assay work that otherwise would not have been possible without adding resources. Larger robotics systems typically take longer to recoup the costs.

Another less common way to justify automated systems is through improved safety, including waste reduction/elimination, reduced exposure to potentially hazardous compounds, solvents, and reagents. The reduction in waste generated is a key aspect to evaluate, especially as the cost of disposal of solvents and chemicals gets more and more expensive. This opportunity is primarily the result of the reduced volumes used during preparation of samples on an automated system. Exposure to solvents and chemical compounds is also becoming more of a concern. More and more new drug entities are highly potent, and prolonged exposure can be harmful. For example, using the homogenizer on the Benchmate TPW to grind tablets in solution greatly is significantly safer than utilizing a mortar and pestle to break up the tablet and then dissolving in solution.

Another opportunity for justifying the purchase of an automated system is better staff utilization. By automating routine and repetitive tasks, analysts are freed up to do more complex and challenging tasks, rather than eliminating their job. Staff utilization and headcount savings was used to help justify several systems in our laboratories during the last few years. Saving of one to two Full-Time Equivalent (FTEs) have been demonstrated by utilizing a Benchmate TPW for content uniformity testing of several projects. Similarly, estimated savings for implementing an automated dissolution system is two to four FTEs.

Automated systems can also be justified on the grounds of better quality data and results. One distinct advantage of automated systems is that processes are performed very uniformly. With this can come improved assay precision and performance. This is especially true when prepared samples are unstable or degrade upon sitting in an autosampler. In one case, a 50% improvement in method variability was realized by transferring the manual analytical method to a Benchmate TPW. In addition to the reduction in method variability, better customer service and reduced cycle time was obtained. Both of these factors can also be used as budget justifications. Reducing laboratory cycle times can have an impact on in-process inventories, provide more timely information to make better process adjustments, etc. Cycle time reductions, which utilimately improves customer service, have been greatly impacted by the use of automation in our labs. Some assays which required several analysts multiple days to perform are now completed in less than one day and require only a single analyst to support the automated system.

### Implementation

The next major challenge for laboratory management is implementation. Just like the project and hardware selection, there are several factors to consider that can impact an implementation plan. The first consideration is resource availability to carry out the implementation. The first question to ask is 'does the necessary expertise and resources exist in-house that can carry out the development, programming, and installation of the automated system or must it be contracted to the vendor or a systems integrator?' The level of expertise required does increase with the complexity of the application. For that reason, it is very important for laboratory management to consider the complexity of the project when developing an implementation strategy. Simpler applications, such as a Benchmate or Benchmate TPW, can usually be implemented in-house with minimal difficulty. However, when it comes to more complex automation such as a robotics system, there are drawbacks to both in-house implementation and contracting it out. A big drawback of in-house development is having the expertise and skills necessary to implement a complex system. Many laboratories, especially those just getting involved with automated systems, will not have the necessary level of expertise, making contracting a more viable option. The big drawback with contracting is less control and understanding of how the system is programmed and components are integrated. As a result, one becomes very dependent on the contractor for troubleshooting problems and making modifications. Such is the case with a new Zymate automated dissolution system being installed in our laboratories. Due to a lack of resources with the necessary expertise and the complexity of the system, as well as an aggressive time line, the only option was to purchase a complete dissolution robotics system from Zymark.

A third consideration that can affect implementation is the time 'line' for completion. Often time lines are very aggressive and as a result do slip to varying degrees. One reason they typically slip is under estimation of the time necessary to come up the learning curve on a new automated system. Coming up the learning curve can range from a few weeks to more than a year and is very dependent again on the complexity of the system.

### Validation

Validation is the next critical step in implementing a successful automated system and represents probably one of the 'newest' challenges for laboratory management. Over the last few years, the importance of equipment and system validation throughout the laboratory has been increasing due to stricter interpretations of GMP guide lines. The increased interest in validation by scientists, vendors, and regulatory agencies reinforces this importance. There are many different approaches to validating automated systems as demonstrated by the numerous papers presented at various conferences during the last three to four years. In general, though, validation can be paired down to a few key aspects. They include installation and operational qualification of critical components, demonstrating acceptable performance characteristics of the system as a whole, as well as developing a strategy to monitor and ensure acceptable performance characteristics of the automated system. This translates into a significant increase in documentation. Consequently, the process of validation can be very time consuming. In the past, validation was very important to ensuring proper implementation of an automated system. Today, with more emphasis being placed on the quality and accuracy of analytical results, validation has become an absolute requirement to guarantee product quality.

### Training and support

The last management challenge is training and support, which is essential to ensuring the success of an automated system and an automation program. Technical training is very important to this success. Many vendors offer training classes for their hardware and systems, but that's just one aspect of technical training. Having the right people with the right background and basic skills first is just as important. For example, we have found that electronic and instrumentation skills are, at times, more important than analytical skills for analysts developing, implementing, and supporting automated laboratory systems. As a result, many of our analysts that support our automated systems have two-year electrical engineering technology degrees, and in many cases have developed into some of our top analytical laboratory analysts, but not without a significant investment in technical training. The same can be said for our instrument repair technicians. We are very fortunate to have in-house instrument maintenance and repair services for many of our analytical instruments and automated systems. This greatly reduces down time due to component failures since they can respond immediately.

Support functions are also very important to the success of an automated system and an automation program. As mentioned previously, vendor support is very important, especially when starting to establish automation in an area. It's probably no surprise that the quality of the support is typically dependent on the quality of the individuals that you interact with. Developing good working relationships with vendors and their representatives is also important, as is their understanding of your direction, goals, and needs so as to better serve you, their customer.

Management support is also very important. Laboratory management, through his or her support, can ensure the success or failure of an automated system and/or program. Laboratory management must provide support to those directly involved with automated systems. This support must come in the form of training, resources, and funds to adequately develop, implement, validate, and support automated systems and the program. Laboratory management can also serve as a 'champion' for laboratory automation, by educating upper management and other areas on the benefits and limitations of laboratory automation, as well as helping them understand the direction the technology is going to ensure future success. In addition, it is important to keep up with current technology and incorporate this into the future direction of his or her area of influence. The 'champion' should also be coach, developing other 'champions', because a true measure of the success of a 'champion' is the continued automation success of an area long after he or she has moved on.

### Conclusions

Laboratory management will continue to be faced with new and different challenges as business focuses and customers change. Expectations to improve laboratory efficiency and productivity, reduce cycle times, improve technical staff utilization, provide a safer work environment, and improve the quality of analytical results will continue to drive the need for laboratory automation. Laboratory management must look for new opportunities to utilize automation as well as serve as a 'champion' of this technology in order to meet these exceptions.

### Acknowledgments

The current laboratory automation status within the Quality Control Laboratories are the result of the efforts of many individuals who have contributed to various projects. The author would like to thank them for their valuable contributions to the success of this automation program: *Benchmate TPW projects:* Doug Allgier—Eli Lilly, Quality Control Laboratories; Tim Houck—Eli Lilly, Quality Control Laboratories; and John Everett—Eli Lilly, Quality Control Laboratories. *Zymate dissolution automation project:* Frank Smietana—Eli Lilly, Quality Control Laboratories; and Bob Houser—Zymark Corporation. *Feed assay automation projects:* Mike Hinshaw—Eli Lilly, Lilly Research Laboratories; Jose Cardenas—Eli Lilly, Quality Control Laboratories; and Chuck Scott—Eli Lilly, Quality Control Laboratories.

### Reference

1. Hinshaw, M. E., Cardenas, J., and Scott, C., ISLAR '94, Boston, MA (October 1994).

### Notes

Performance Excellence is a registered trademark of Eli Lilly and Company, Florida Power and Light Company, and Organizational Dynamics, Inc.

Benchmate, Benchmate TWP, Zymate, and Zymark are registered trademarks of Zymark Corporation.

Micro Robot is a registered trademark of Mitsubishi Electronics Corporation.

---

The following ISLAR papers will appear in the next available issue.

*Managing laboratory automation* by T. J. Saboe.
*Reality in the 1990s and beyond—more with less* by P. A. Lane.
*The human side of automation: experience in clinical pharmacology* by J. R. Powell.

